# Study of the Impact of Chronic Obstructive Pulmonary Disease on Vocal Function

**DOI:** 10.1055/s-0045-1811696

**Published:** 2025-11-11

**Authors:** Omnia Zakaria Elshebl, Asmaa Eldesouky Mohamed, Salwa Ahmed Abdelhay Ahmed

**Affiliations:** 1 Department of Phoniatrics and Otorhinolaryngology, Faculty of Medicine, Benha University, Benha, Egypt; 2 Department of Chest Diseases, Faculty of Medicine, Benha University, Benha, Egypt

**Keywords:** COPD, voice quality, pulmonary function, acoustic analysis, GOLD classification

## Abstract

**Introduction:**

Chronic obstructive pulmonary disease (COPD) affects lung function, but its impact on vocal function remains understudied.

**Objective:**

To investigate the relationship involving COPD severity and vocal acoustic parameters and to assess the correlations regarding acoustic measures and pulmonary function tests in clinically stable COPD patients.

**Methods:**

The present observational study included 40 COPD patients diagnosed according to the 2023 guidelines of the Global Initiative for Chronic Obstructive Lung Disease (GOLD). All patients underwent spirometry, an otorhinolaryngological examination, and acoustic voice analysis, including fundamental frequency (F0), harmonics-to-noise ratio (HNR), jitter, and shimmer. The Arabic version of the Voice-Related Quality of Life (V-RQOL) questionnaire was used to assess voice-related impairment.

**Results:**

The sample had a mean age of 65 ± 6 years, with 92.5% of male subjects. Dysphonia was observed in 57.5% of the patients. Laryngoscopic findings included Reinke's edema (42.5%), laryngitis (25%), polypoid degeneration (20%), and phonasthenia (12.5%). The acoustic analysis revealed significant negative correlations involving pulmonary function parameters and vocal measures. The regression analysis demonstrated forced expiratory volume in the first second (FEV1) and FEV1/forced vital capacity (FVC) as significant predictors of mean pitch, jitter, and shimmer, with each unit increase in FEV1 associated with a decrease in these acoustic measures ( *p* < 0.001).

**Conclusion:**

The severity of COPD is significantly associated with acoustic voice alterations, with disease progression leading to measurable impairments in vocal function. These findings highlight the importance of voice assessment in COPD management.

## Introduction

Chronic obstructive pulmonary disease (COPD) is an intricate respiratory disorder characterized by a persistent limitation to airflow caused by chronic bronchitis or emphysema. 1 2 The main factor contributing to this illness is long-term exposure to harmful gases or particulate matter, typically stemming from the inhalation of cigarette smoke. Patients with COPD undergo a deterioration in their quality of life and an increase in morbidity and mortality rates, rendering this condition a significant public health concern. As COPD progresses, it not only impacts the lungs but also impairs other aspects of health, including vocal communication. 3 An estimated 392 million people are living with COPD, with 75% of them in low- and middle-income countries. 4

Voice dysfunction is an often overlooked yet significant complication of COPD. The relationship between COPD and vocal impairment involves a complex interplay between respiratory limitations and vocal mechanics. 5 6 The voice is powered by airflow from the lungs, and any condition that compromises pulmonary function can adversely affect phonation. Specifically, COPD-related changes in lung mechanics can lead to alterations in voice quality, which may manifest as hoarseness, reduced vocal intensity, and increased vocal fatigue. 7 8 These vocal impairments can further degrade the patients' quality of life, affecting their social interactions and psychological well-being.

Despite the prevalence of voice disorders among COPD sufferers, there is limited research directly investigating the pathophysiological mechanisms linking reduced pulmonary function to vocal dysfunction.

The present study hypothesizes that deteriorations in pulmonary function, as evidenced by pulmonary function tests (PFTs), are closely associated with adverse changes in voice quality in COPD patients. The study aims to investigate the correlation involving COPD severity and vocal acoustic parameters and to assess the correlations regarding acoustic parameters and PFTs in clinically stable COPD patients.

## Methods

### Design and Population

The current is an observational study with 40 clinically stable COPD patients who underwent follow-up in the Department of Chest Diseases and Chest Outpatient Clinic of Benha University Hospital from February 2023 to August 2023. The study was conducted after we obtained approval (no. RC 23-2-2023) from the Institutional Review Board (IRB) of the Faculty of Medicine of Benha University. Written consent was obtained from all patients included.

### Diagnosis and Severity of COPD

The diagnosis and severity of COPD were determined according to the 2023 guidelines of the Global Initiative for Chronic Obstructive Lung Disease (GOLD). 9 The COPD patients included in the study were diagnosed based on their history, clinical examination, and spirometry results (forced expiratory volume in the first second [FEV1]/forced vital capacity [FVC] ratio < 70%). Disease severity was classified according to postbronchodilator FEV1 into mild, moderate, severe, and very severe COPD. Stage 1 (mild) was defined as a postbronchodilator FEV1 ≥ 80% predicted, stage 2 (moderate), as a postbronchodilator FEV1 between 50% and 80% predicted, stage 3 (severe), as a postbronchodilator FEV1 between 30% and 50% predicted, and stage 4 (very severe), as a postbronchodilator FEV1 < 30% predicted. 9

### Patient Assessments

All patients underwent a comprehensive assessment that included history taking (age, gender, drug history, and smoking index), a local chest examination, and a general examination. A chest X-ray was performed, along with PFTs (spirometry).

Spirometry was conducted in the PFT Unit at Benha University Hospital using a computed pulmonary function apparatus (Jaeger Master Screen PFT, CareFusion UK Ltd). The spirometry measured slow vital capacity, FVC, pre- and postbronchodilator FEV1, and the FEV1/FVC ratio. The reversibility test measured FEV1 before and after the administration of 400 µg of short-acting beta2-agonist, with FEV1 measured 10 to 15 minutes after administration. Reversibility was defined as a change in FEV1 by ≥ 12% and ≥ 200 mL from baseline, according to the American Thoracic Society/European Respiratory Society (ATS/ERS) 2019 guidelines. 10 Maximum voluntary ventilation (MVV) was also measured. Patients were diagnosed with COPD if their postbronchodilator FEV1/FVC ratio was < 0.70.

### ENT and Acoustic Analysis

The ear, nose, and throat (ENT) examination included an assessment of the oral cavity, tongue, soft and hard palates, pharynx, and larynx. An endoscopic examination of the larynx was conducted using an indirect laryngoscope and camera (KARL STORZ SE & Co. KG) to assess any vocal fold pathologies. Acoustic analysis of the voice was performed, measuring the fundamental frequency (F0), jitter, shimmer, and the harmonics-to-noise ratio (HNR). Jitter was measured using the Jitter Percent (Jitt) and Absolute Jitter (Jitta) parameters, while shimmer was assessed using the Shimmer Percent (Shim) and Shimmer in Decibels (ShdB) parameters.

The voice samples were recorded during sustained phonation of the vowel /a/ at a comfortable pitch and loudness. The maximum phonation time (MPT/a/) was used, and a time window of 3 seconds was selected for analysis.

Two experienced phoniatricians conducted all measurements in a double-blinded manner using the Praat software (free and open source). The findings were approved if there was agreement between the two observers. AKG 190HS microphone (Samsung Electronics) was placed at a distance of 30 cm to record the patient's voice.

### Voice-Related Quality of Life (V-RQOL)

Voice-related quality of life (V-RQOL) was assessed using the Arabic version of the V-RQOL questionnaire to detect the effect of COPD on voice 11 ( Appendix 1 ). The questionnaire consists of 10 questions, each with a score ranging from 1 (no problem) to 5 (very severe problem). The total score is calculated by adding the scores on each question.

### Statistical Methods

The data was managed and statistically analyzed using the IBM SPSS Statistics for Windows (IBM Corp.) software, version 28.0. The normality of the quantitative data was evaluated using the Shapiro-Wilk test and direct data visualization techniques. As per the concept of normalcy, the quantitative data were expressed as mean and standard deviation (SD) values. The categorical data were expressed as numerical values and percentages. Pearson's correlation was used to evaluate the associations involving acoustic measurements and PFTs. The acoustic measures of the patients who used and did not use steroids were compared through the independent samples *t* -test. A multivariate linear regression analysis was conducted to forecast various acoustic measurements based on the PFTs. The regression coefficients were computed together with their corresponding 95%CIs. The statistical tests conducted were all two-sided. Significance was set at *p* < 0.05.

## Results

### General Characteristics

**Fig. 1:**
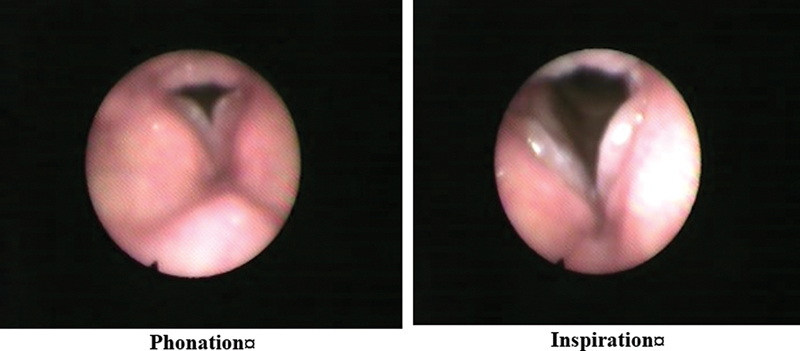
Polypoid degeneration.

The mean age of the participants was of 65 ± 6 years. Most participants were males, comprising 92.5% (n = 37), and the rate of female subjects was of7.5% (n = 3). Smoking history revealed that 95% (n = 38) of the participants were past smokers, and 5% (n = 2) were non-smokers. Dysphonia was present in 57.5% (n = 23) of the participants. The laryngoscopic examination identified various conditions: Reinke's edema in 42.5% (n = 17), laryngitis in 25% (n = 10), polypoid degeneration in 20% (n = 8), and phonasthenia in 12.5% (n = 5) ( Fig. 1 2 3 ). The mean total quality of voice (QOV) score was of 26 ± 8. The severity of the disease according to the GOLD classification, showed that 27.5% (n = 11) presented mild disease, 35% (n = 14), moderate disease, 22.5% (n = 9), severe disease, and 15% (n = 6), very severe disease ( Table 1 ).

**Fig. 2:**
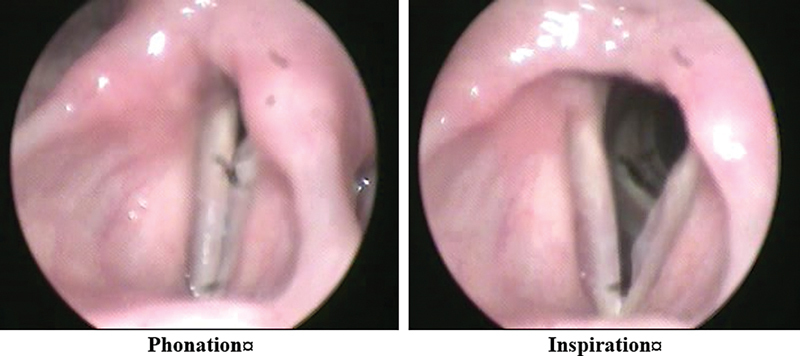
Rienek's edema.

**Fig. 3:**
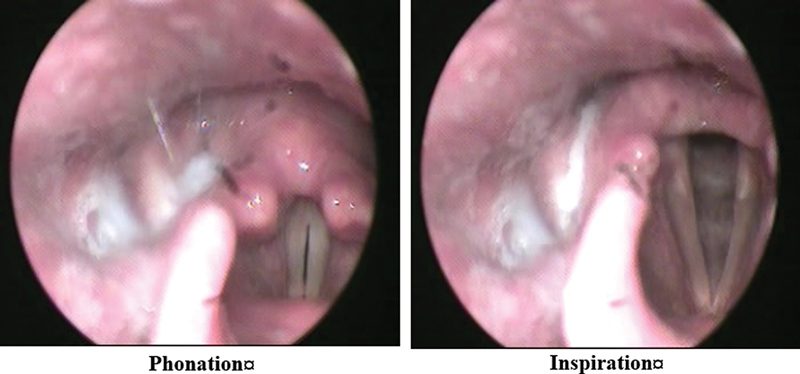
Phonasthenia.

**Table 1:** General characteristics of the study sample

General characteristics		
**Mean age (years)**		65 ± 6
**Gender: n (%)**		
Male		37 (92.5)
Female		3 (7.5)
**Smoking: n (%)**		
Past smoker		38 (95)
Non-smoker		2 (5)
**Dysphonia: n (%)**		23 (57.5)
**Laryngoscopic findings: n (%)**		
Reinek''s edema		17 (42.5)
Laryngitis		10 (25)
Polypoid degeneration		8 (20)
Phonasthenia		5 (12.5)
**Mean total Quality of Voice score**		26 ± 8
**GOLD classification: n (%)**		
Mild disease		11 (27.5)
Moderate disease		14 (35)
Severe disease		9 (22.5)
Very severe		6 (15)
**Inhaled steroid: n (%)**		15 (37.5)

**Abbreviation:** GOLD, Global Initiative for Chronic Obstructive Lung Disease.

### Acoustic Measures and Pulmonary Function Tests

The mean pitch recorded was of 267.26 ± 115.3 Hz. The mean jitter was of 3.46 ± 1.38%, while the mean shimmer was of 1.47 ± 0.21%. The mean HNR was of 3.66 ± 1.34. The PFTs revealed a mean FEV1 of 57 ± 19%. The mean FVC was of 79 ± 14%. And the mean FEV1/FVC was of 52 ± 15% ( Table 2 ).

**Table 2:** Acoustic parameters and pulmonary functions tests

	Mean ± standard deviation
**Mean** **pitch**	267.26 ± 115.3
**Jitter**	3.46 ± 1.38
**Shimmer**	1.47 ± 0.21
**Harmonics-to-noise ratio**	3.66 ± 1.34
**FEV1 (%)**	57 ± 19
**FVC (%)**	79 ± 14
**FEV1/FVC ratio (%)**	52 ± 15

**Abbreviations:** FEV1, forced expiratory volume in the first second (FEV1); FVC, forced vital capacity.

### Correlations Involving Acoustic Parameters and Pulmonary Function Tests

**Fig. 4:**
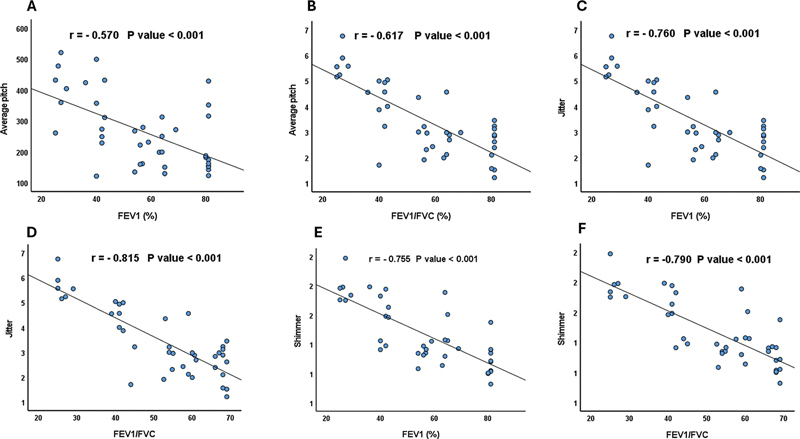
Correlation involving ( **A** ) mean pitch and forced expiratory volume in the first second (FEV1); ( **B** ) average pitch and FEV1/forced vital capacity (FVC); ( **C** ) jitter and FEV1; ( **D** ) jitter and FEV1/FVC; ( **E** ) shimmer and FEV1; and ( **F** ) shimmer and FEV1/FVC.

The correlations involving various vocal and pulmonary function parameters were analyzed. Significant negative correlations were observed between FEV1 (%) and several vocal parameters: mean pitch (r = −0.570; *p* < 0.001), jitter (r = −0.760; *p* < 0.001), and shimmer (r = −0.755; *p* < 0.001). Similarly, significant negative correlations were found between the FEV1/FVC (%) and the mean pitch (r = −0.617; *p* < 0.001), jitter (r = −0.815; *p* < 0.001), and shimmer (r = −0.790; *p* < 0.001) ( Table 3 ; Fig. 4 ). The GOLD classification showed significant positive correlations with the mean pitch (r = 0.594; *p* < 0.001), jitter (r = 0.787; *p* < 0.001), and shimmer (r = 0.793; *p* < 0.001) ( Table 3 ).

**Table 3:** Correlation between acoustic parameters and pulmonary function tests

	Mean pitch		Jitter		Shimmer		HNR		Total QOV	
	r	*p*	r	*p*	r	*p*	r	*p*	r	*p*
**FEV1 (%)**	−0.570	< 0.001*	−0.760	< 0.001*	−0.755	< 0.001*	−0.081	0.618	0.004	0.981
**FVC (%)**	−0.104	0.522	−0.258	0.108	−0.079	0.63	0.259	0.107	−0.093	0.569
**FEV1/FVC ratio (%)**	−0.617	< 0.001*	−0.815	<0.001*	−0.790	< 0.001*	−0.103	0.527	−0.019	0.909
**GOLD classification**	0.594	< 0.001*	0.787	< 0.001*	0.793	< 0.001*	0.074	0.649	0.046	0.78

**Abbreviations:** FEV1, forced expiratory volume in the first second (FEV1); FVC, forced vital capacity; GOLD, Global Initiative for Chronic Obstructive Lung Disease; HNR, harmonics-to-noise ratio; QOV, quality of voice; r, correlation coefficient.

**Note:** *Statistically significant .

### Acoustic Measures according to Steroid Use

In the current study, inhaled steroids significantly influenced several voice parameters. Patients using inhaled steroids presented a higher mean pitch (344.3 ± 110.16) compared to those not using inhaled steroids (221.03 ± 92.67), with a highly significant difference ( *p* < 0.001). Similarly, jitter was significantly elevated in the inhaled steroid group (mean = 4.64 ± 1.28) compared to the non-user group (mean = 2.75 ± 0.86), also showing a highly significant difference ( *p* < 0.001). Moreover, shimmer levels were higher in the inhaled-steroid group (mean = 1.67 ± 0.17) compared to non-users (mean = 1.35 ± 0.14), with this difference being statistically significant ( *p* < 0.001). However, the HNR was insignificantly different between the two studied groups ( *p* = 0.492).

### Prediction of Acoustic Measures

A multivariate linear regression analysis was performed to predict different acoustic measures based on the PFTs. Only variables that showed significant correlations on the univariate level were included. Separate regression models were used to avoid potential multicollinearity; each adjusted for age and gender ( Table 4 ).

**Table 4:** Multivariate linear regression analysis to predict different acoustic measures from pulmonary function tests

	Mean pitch		Jitter		Shimmer	
	B (95%CI) ^†^	*p*	B (95%CI) ^†^	*p*	B (95%CI) ^†^	*p*
**FEV1 (%)**	−3.391 (−5.022–−1.76)	< 0.001*	−0.054 (−0.069 –−0.038)	< 0.001*	−0.008 (−0.011–−0.006)	< 0.001*
**FEV1/FVC**	−4.84 (−6.886–−2.793)	< 0.001*	−0.076 (−0.094–−0.058)	< 0.001*	−0.012 (−0.014–−0.009)	< 0.001*

**Abbreviations:** B, regression coefficient; FEV1, forced expiratory volume in the first second (FEV1); FVC, forced vital capacity.

**Notes:** *Statistically significant; ^†^ adjusted for age and gender.

The model revealed that, controlling age and gender, FEV1 was a significant predictor of the mean pitch, jitter, and shimmer. One unit increase in the FEV1 was associated with: a decrease in mean pitch by 3.391 (B = −3.391; 95%CI = −5.022–−1.76; *p* < 0.001), a decrease in jitter by 0.054 (B = −0.054; 95%CI = −0.069–−0.038; *p* < 0.001), and a decrease in shimmer by 0.008 (B = −0.008; 95%CI = −0.011–−0.006; *p* < 0.001) ( Table 4 ).

Similarly, controlling for age and gender, the FEV1/FVC was a significant predictor of the mean pitch, jitter, and shimmer. One unit increase in the FEV1/FVC was associated with: a decrease in the mean pitch by 4.84 (B = −4.84; 95%CI = −6.886–−2.793; *p* < 0.001), a decrease in jitter by 0.076 (B = −0.076; 95%CI = −0.094–−0.058; *p* < 0.001), and a decrease in shimmer by 0.012 (B = −0.012; 95%CI = −0.014–−0.009; *p* < 0.001) ( Table 4 ).

## Discussion

A common respiratory condition that causes ongoing restriction of airflow, COPD is linked to substantial illness and death. 12 Although the effects of COPD on the lungs are well-known, its impact on voice quality is a field of growing interest. Voice disorders in COPD patients can greatly affect their quality of life, influencing their social interactions and emotional state. 13 The goal of the present study is to investigate the relationship between the severity of COPD and voice quality by assessing vocal parameters and correlating them with PFTs.

To the best of our knowledge, the current is one of the first studies to empirically quantify the relationship between the severity of pulmonary dysfunction and its direct impact on vocal quality in COPD patients. Previous studies 7 have primarily focused on the broader symptomatic impacts of COPD, including general dysphonia and voice fatigue, without delving into the detailed acoustic properties that could be quantitatively linked to specific pulmonary function metrics.

In the present work, the patients had a mean age of 65 ± 6 years, most participants were male (92.5%; n = 37), 95% (n = 38) were past smokers, and 57.5% (n = 23) of them presented dysphonia. In line with our findings, Saeed et al. 14 also found significant voice impairments in COPD patients: they identified that 30% of COPD patients exhibited various dysphonia grades and impaired voice quality, and they also reported structural changes such as diffuse congestion and unhealthy mucosa in 36.6% of COPD patients.

In agreement with our results, Shastry et al. 15 observed notable variations in acoustic and perceptual voice characteristics among COPD patients when compared to healthy cases. Their investigation found that there were decreases in the fundamental frequency and in the frequency range, as well as increases in pitch and amplitude perturbation measures, and in noise measures.

Smoking greatly worsens the decline in lung function and can cause dysphonia. Tobacco-related hoarseness can be caused by laryngeal or vocal-fold illnesses, such as inflammation or tumor formation. Matsuo et al. 16 discovered that tobacco users who experience hoarseness have a higher occurrence of polypoid vocal fold lesions and head and neck cancer. Furthermore, research has documented immediate and long-term impacts of cigarette smoke on the functioning of the oropharynx. Dua et al. 17 conducted a study comparing the contractile reflexes of the pharyngo-upper esophageal sphincter in healthy smokers and non-smokers. The results showed that smokers had a larger threshold for initiating this reflex and a higher occurrence of gastroesophageal reflux disease (GERD), microaspiration, and hoarseness.

In agreement with our results, Mohamed and El maghraby 5 reported significant voice changes in COPD patients, noting that dysphonia was perceived in 50% of their participants. Their patients were former or current smokers, and they found significant positive correlations involving the smoking index and acoustic measures such as Jitt and Shim, as well as the grade of dysphonia. Santos et al. 8 reported a direct correlation between the duration of the smoking habit and alterations in vocal production, including increased vocal tension, roughness, breathiness, and instability. This finding is consistent with our results, since 38 (95%) of our participants were past smokers, and a significant proportion exhibited dysphonia (23; 57.5%). Similar correlations were found by Tajada et al., 18 reinforcing the impact of smoking on vocal health.

In the current work, the acoustic measures showed a mean pitch of 267.26 ± 115.3 Hz, a mean jitter of 3.46 ± 1.38%, a mean shimmer of 1.47 ± 0.21%, and a mean HNR of 3.66 ± 1.34, while the PFTs revealed a mean FEV1 of 57 ± 19%, a mean FVC of 79 ± 14%, and a mean FEV1/FVC of 52 ± 15%. Our results are in line with those of the acoustic analysis by Saeed et al., 14 which showed increased jitter (mean = 2.02 ± 0.20%) and shimmer (mean = 1.06 ± 0.24%) and a decreased HNR (mean = 14.00 ± 0.16).

Our analysis revealed significant negative correlations involving the FEV1 and several vocal parameters, including mean pitch, jitter, and shimmer, as well as regarding the FEV1/FVC and these same vocal parameters, while the GOLD classification showed significant positive correlations with mean pitch, jitter, and shimmer.

The observed strong associations involving the FEV1, FEV1/FVC, and voice characteristics such as jitter and shimmer are consistent with previous research 5 6 indicating that respiratory conditions have an impact on vocal production. Reduced FEV1 and FEV1/FVC are commonly indicative of more severe obstructive patterns. In our study, we observed that these patterns were related with poorer voice quality, as demonstrated by greater levels of jitter and shimmer. This connection can be elucidated by examining the mechanics of voice production, in which a steady and adequate pulmonary output is essential to sustain consistent vocal fold vibrations. When there is a decline in lung function, as often observed in COPD, the airflow required to produce these vibrations is impaired. This results in uneven movements of the vocal folds and, subsequently, the acoustic abnormalities identified in the current study.

Vocal production depends on a stable respiratory system that generates adequate subglottic pressure to facilitate vocal fold vibration. In COPD, the airflow limitation caused by airway obstruction, lung hyperinflation, and expiratory flow restriction reduces breath support for phonation, leading to irregular vocal fold oscillations. 19 20 This disruption is reflected in increased jitter and shimmer values, indicating greater frequency and amplitude instability in voice production. The strong negative correlations regarding the FEV1 and these acoustic measures suggest that, as pulmonary function deteriorates, vocal fold control becomes more erratic.

The reduction in the HNR further supports this finding, as a lower HNR indicates increased turbulent airflow due to incomplete glottal closure or irregular vocal fold vibrations. Respiratory muscle fatigue and air trapping related to COPD contribute to inefficient phonation, leading to increased breathiness and spectral noise in the voice signal. Additionally, chronic inflammation, mucus hypersecretion, and laryngeal structural changes—which are common in COPD patients, particularly smokers—can further impair vocal function by inducing edema, vocal-fold thickening, and reduced mucosal pliability. 21

These physiological and anatomical alterations collectively explain the observed acoustic deviations, reinforcing our hypothesis that worsening pulmonary function is directly linked to voice instability. The significant correlations involving spirometry parameters and vocal measures highlight the impact of COPD severity on vocal quality, with potential consequences for communication and overall quality of life. Given the limited research in this field, the current study provides novel insights into the interaction between pulmonary dysfunction and vocal mechanics, underscoring the importance of incorporating voice assessment into COPD management.

Comparably, Saeed et al. 14 found significant correlations between the severity of COPD and voice quality. A strong and statistically significant link was found between the severity of spirometry and the dysphonia grade and the type of voice in patients with bronchial asthma. Shastry et al. 15 noted a significant negative impact of COPD on voice measures, with COPD patients showing increased noise measures and higher NHR, indicating reduced subglottal pressure, increased breathiness, and irregularity in vocal fold vibration.

In agreement with our results, Silva et al. 13 found that V-RQOL in COPD patients is significantly impacted by the disease. Their study identified a moderate, negative correlation between the V-RQOL scores and the COPD Assessment Test (CAT) scores, indicating that, as COPD severity increases, V-RQOL diminishes. Okutan et al. 22 found that dyspnea significantly impacts the quality of life of COPD patients, as assessed by the CAT score. Their study showed a positive correlation between CAT scores and dyspnea scale scores ( *p* < 0.001), as well as between CAT scores and the GOLD stage of COPD ( *p* < 0.001). Zeng et al. 23 examined the acoustic and prosodic properties of a lung function-sensitive repeated-word speech articulation test. They reported that ambiguous auditory metrics, such as increasing pause ratios, may indicate respiratory diseases. We also found higher jitter and shimmer and lower HNR in COPD patients, indicating poor vocal fold vibration and respiratory mechanics. Zeng et al. 23 also revealed the importance of the inclusion of speech breathing and prosodic aspects in PFTs, highlighting their potential as non-invasive respiratory health indicators.

Regarding acoustic measures according to steroid use in the present study, inhaled steroid use was significantly associated with higher mean pitch and increased jitter and shimmer. Consistently, Mohamed and El maghraby 5 identified significant positive correlations involving the use of large doses of inhaled corticosteroids and increased Jitt, Shim, and grade of dysphonia.

In the current study, multivariate linear regression analysis was performed to predict different acoustic measures based on PFTs. The analysis revealed the FEV1 and FEV1/FVC as significant predictors of mean pitch, jitter, and shimmer, with each unit increase in FEV1 associated with a decrease in these acoustic measures.

The present study has several limitations that should be considered. First, the limited sample size may restrict the generalizability of our results. Second, we did not examine how various inhaler devices affect voice quality, which could influence acoustic characteristics. Additionally, the study only included clinically stable COPD patients, thus it did not account for disease stability or exacerbations, which might alter voice quality. Finally, the cross-sectional design provides a snapshot of COPD severity and voice quality, but longitudinal studies are needed to analyze changes over time and the COPD long-term effects on vocal health.

## Conclusion

Deterioration in lung function among COPD patients is significantly associated with measurable changes in voice quality. As pulmonary function declines, marked by decreases in the FEV1 and FEV1/FVC, vocal abnormalities such as increased jitter and shimmer intensify, indicating compromised vocal fold vibration and stability.
