# Supplementary material for: Study of the Impact of Chronic Obstructive Pulmonary Disease on Vocal Function
Source: Int Arch Otorhinolaryngol. 2025 Nov 11;29(4):1–8. doi: 10.1055/s-0045-1811696 (PMC12614902; doi:10.1055/s-0045-1811696)
Supplement: Supplementary file 1 — Supplementary Material [file 10-1055-s-0045-1811696-s241831.pdf]

Appendix 1

Arabic Version of the Voice-Related Quality of Life (V-RQOL) questionnaire

-الاسم:

- التاريخ:

➢استخدم المقياس التالي لتصنيف مقدار المشكلة:

1 = لا يوجد او ليست مشكلة

2 = بقدر صغير(قليل)

3 =بقدر معتدل (متوسط)

4 = بقدر كثير(كبير)

5 = المشكلة "سيئة" كما يمكن أن تكون

5	4	3	2	1	بسبب صوتي	
					أجد صعوبة في التحدث بصوت عال او صعوبة في سماع صوتي في المواقف الصاخبة	1
					ينفذ مني الهواء وأحتاج إلى التنفس بشكل متكرر عندما أتكلم.	2
					في بعض الأحيان لا أعرف ماذا سيحدث عندما أبدأ في الكلام.	3
					أنا في بعض الأحيان أشعر بالقلق أو الإحباط (بسبب صوتي).	4
					أحيانًا أشعر بالاكتئاب (بسبب صوتي).	5
					أجد مشكلة في استخدام الهاتف (بسبب صوتي).	6
					أجد صعوبة في القيام بعملي أو ممارسة مهنتي (بسبب صوتي).	7
					أتجنب الخروج اجتماعيا (بسبب صوتي).	8
					علي أن أكرر كلامي لأكون مفهوماً.	9
					لقد أصبحت أقل خروجا (انفتاحا) (بسبب صوتي).	10

 © 2025. The Author(s).
